# 4-(4-Nitro­benz­yl)pyridinium 5-nitro­salicylate

**DOI:** 10.1107/S1600536810014698

**Published:** 2010-04-24

**Authors:** Graham Smith, Urs D. Wermuth

**Affiliations:** a Faculty of Science and Technology, Queensland University of Technology, GPO Box 2434, Brisbane, Queensland 4001, Australia

## Abstract

In the title salt, C_12_H_11_N_2_O_2_^+^·C_7_H_4_NO_5_^−^, the cations and anions inter­act through asymmetric cyclic pyridinium–carboxyl­ate N—H⋯O,O′ hydrogen-bonding associations [graph set *R*_1_^2^(4)], giving discrete heterodimers having weak cation–anion π–π aromatic ring inter­actions [minimum ring centroid separation = 3.7116 (9) Å].

## Related literature

For structural data on nitro-substituted 4-benzyl­pyridines and related compounds, see Seff & Trueblood (1968 ▶); Ottersen & Seff (1974 ▶); Scherl *et al.* (1996 ▶); Smith *et al.* (1997 ▶); Naumov *et al.* (2002 ▶). For structures of Lewis base salts of 5-nitro­salicylic acid, see: Smith *et al.* (1996 ▶, 2005 ▶, 2006 ▶). For graph-set motifs, see: Etter *et al.* (1990 ▶).
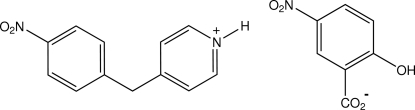


## Experimental

### 

#### Crystal data

C_12_H_11_N_2_O_2_^+^·C_7_H_4_NO_5_^−^
*M* *_r_* = 397.34
Triclinic, 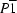

*a* = 8.3287 (5) Å
*b* = 10.8219 (7) Å
*c* = 11.3896 (8) Å
α = 65.160 (6)°
β = 88.286 (5)°
γ = 70.553 (6)°
*V* = 871.17 (12) Å^3^
*Z* = 2
Mo *K*α radiation
μ = 0.12 mm^−1^
*T* = 200 K
0.25 × 0.25 × 0.20 mm

#### Data collection

Oxford Diffraction Gemini-S CCD-detector diffractometer
Absorption correction: multi-scan (*SADABS*; Sheldrick, 1996 ▶) *T*_min_ = 0.795, *T*_max_ = 0.900
10797 measured reflections
3419 independent reflections
2615 reflections with *I* > 2σ(*I*)
*R*_int_ = 0.021

#### Refinement

*R*[*F* ^2^ > 2σ(*F* ^2^)] = 0.034
*wR*(*F* ^2^) = 0.091
*S* = 1.03
3419 reflections
270 parameters
H atoms treated by a mixture of independent and constrained refinement
Δρ_max_ = 0.15 e Å^−3^
Δρ_min_ = −0.19 e Å^−3^

### 

Data collection: *CrysAlis PRO* (Oxford Diffraction, 2009 ▶); cell refinement: *CrysAlis PRO*; data reduction: *CrysAlis PRO*; program(s) used to solve structure: *SHELXS97* (Sheldrick, 2008 ▶); program(s) used to refine structure: *SHELXL97* (Sheldrick, 2008 ▶) within *WinGX* (Farrugia, 1999 ▶); molecular graphics: *PLATON* (Spek, 2009 ▶); software used to prepare material for publication: *PLATON*.

## Supplementary Material

Crystal structure: contains datablocks global, I. DOI: 10.1107/S1600536810014698/pv2273sup1.cif

Structure factors: contains datablocks I. DOI: 10.1107/S1600536810014698/pv2273Isup2.hkl

Additional supplementary materials:  crystallographic information; 3D view; checkCIF report

## Figures and Tables

**Table 1:** Hydrogen-bond geometry (Å, °)

*D*—H⋯*A*	*D*—H	H⋯*A*	*D*⋯*A*	*D*—H⋯*A*
N1—H1⋯O11*A*	0.97 (2)	1.612 (19)	2.5804 (16)	180 (2)
N1—H1⋯O12*A*	0.97 (2)	2.55 (2)	3.1464 (18)	120.2 (14)
O2*A*—H2*A*⋯O12*A*	1.00 (2)	1.48 (2)	2.4623 (17)	165 (2)

**Fig. 1.**
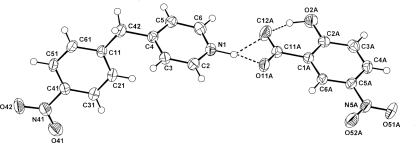
Molecular configuration and atom naming scheme for the hydrogen-bonded NBP cation and 5-NSA anion species in (I). Hydrogen bonds are shown as dashed lines and displacement ellipsoids are drawn at the 50% probability level.

**Fig. 2.**
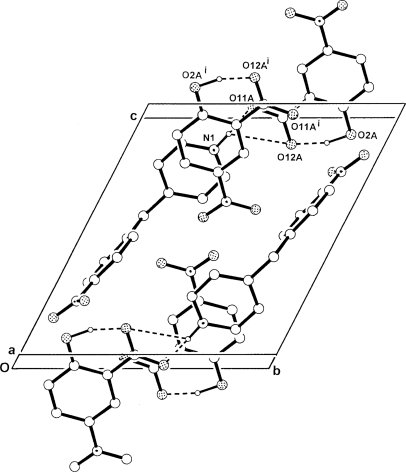
The packing of (I) in the unit cell viewed down the a axis showing partial overlap of cation and anion aromatic rings. Non-associative H atoms are omitted. For symmetry code (i) -x + 1, -y + 1, -z + 2.

## supplementary crystallographic information

### Comment

The Lewis base 4-(4-nitrobenzyl)pyridine (NBP) is an analogue of 2-(2,4-dinitrobenzyl)pyridine (DNBP) which is significant because of its unusual photochromic behaviour in the solid state, although NBP does not possess such properties. The structure of DNBP has been determined (Seff & Trueblood, 1968; Scherl *et al.*, 1996; Naumov *et al.*, 2002), as well that of its isomer 4-(2,4-dinitrobenzyl)pyridine (Ottersen & Seff (1974), but the structure of NBP itself is not known. A structure of a cocrystal adduct of NBP with 4-aminobenzoic acid has been reported (Smith *et al.*, 1997).

Our reaction of NBP with 5-nitrosalicylic acid (5-NSA) gave the title compound C_12_H_11_N_2_O_2_^+^ C_7_H_4_NO_5_^-^ (I), the structure of which is reported here. The acid 5-NSA has proved useful for formation of crystalline salts with a number of Lewis bases (Smith *et al.*, 1996; Smith *et al.*, 2005; Smith *et al.*, 2006). With the title compound (I) (Fig. 1), the NBP cations and 5-NSA anions interact through asymmetric cyclic N^+^–H···O,O'_carboxyl_ hydrogen-bonding associations (Table 1), giving discrete heterodimers [graph set *R*^2^_1_(4) (Etter *et al.*, 1990)]. There are weak cation–anion π–π aromatic ring interactions present in the crystal packing [minimum ring centroid separation between rings N1–C6 and C1A–C6A, 3.7166 (9) Å] (Fig. 2). With the NBP cation the two rings are approximately normal to each other [torsion angle C3–C4–C42–C11, -77.72 (17)°] with the nitro group close to coplanar with the benzene ring [torsion angle C31–C41–N41–O42, 170.27 (13)°]. The usual intramolecular phenolO—H···O_carboxyl_ hydrogen bond [2.4623 (17) Å] is present in the 5-NSA anion which, including the nitro group is close to planar [torsion angles C2A–C1A–C11A–O11A, -173.72 (13)°; C4A–C5A–N5A–O52A, 177.68 (13)°].

### Experimental

The title compound was synthesized by heating together under reflux for 10 minutes, 1 mmol quantities of 4-(4-nitrobenzyl)pyridine with 5-nitrosalicylic acid in 50 ml of 50% ethanol–water. After concentration to *ca.* 30 ml, partial room temperature evaporation of the hot-filtered solution gave yellow crystal aggregates (m.p. 416–417 K) from which a block section was cleaved for the X-ray analysis.

### Refinement

Hydrogen atoms involved in hydrogen-bonding interactions were located by difference methods and their positional and isotropic displacement parameters were refined. The other H-atoms were included in the refinement at calculated positions [C–H = 0.93 Å (aromatic) and 0.97 Å (aliphatic) and U_iso_(H) = 1.2U_eq_(C)], using a riding-model approximation.

### Crystal data

**Table:**

C_12_H_11_N_2_O_2_^+^·C_7_H_4_NO_5_^−^	*Z* = 2
*M**_r_* = 397.34	*F*(000) = 412
Triclinic, *P*1	*D*_x_ = 1.515 Mg m^−^^3^
Hall symbol: -P 1	Melting point = 416–417 K
*a* = 8.3287 (5) Å	Mo *K*α radiation, λ = 0.71073 Å
*b* = 10.8219 (7) Å	Cell parameters from 5316 reflections
*c* = 11.3896 (8) Å	θ = 3.5–27.3°
α = 65.160 (6)°	µ = 0.12 mm^−^^1^
β = 88.286 (5)°	*T* = 200 K
γ = 70.553 (6)°	Block, yellow
*V* = 871.17 (12) Å^3^	0.25 × 0.25 × 0.20 mm

### Data collection

**Table:**

Oxford Diffraction Gemini-S CCD-detector diffractometer	3419 independent reflections
Radiation source: Enhance (Mo) X-ray source	2615 reflections with *I* > 2σ(*I*)
graphite	*R*_int_ = 0.021
ω scans	θ_max_ = 26.0°, θ_min_ = 3.5°
Absorption correction: multi-scan (*SADABS*; Sheldrick, 1996)	*h* = −10→10
*T*_min_ = 0.795, *T*_max_ = 0.900	*k* = −13→13
10797 measured reflections	*l* = −14→14

### Refinement

**Table:**

Refinement on *F*^2^	Primary atom site location: structure-invariant direct methods
Least-squares matrix: full	Secondary atom site location: difference Fourier map
*R*[*F*^2^ > 2σ(*F*^2^)] = 0.034	Hydrogen site location: inferred from neighbouring sites
*wR*(*F*^2^) = 0.091	H atoms treated by a mixture of independent and constrained refinement
*S* = 1.03	*w* = 1/[σ^2^(*F*_o_^2^) + (0.0545*P*)^2^] where *P* = (*F*_o_^2^ + 2*F*_c_^2^)/3
3419 reflections	(Δ/σ)_max_ = 0.001
270 parameters	Δρ_max_ = 0.15 e Å^−^^3^
0 restraints	Δρ_min_ = −0.19 e Å^−^^3^

### Special details

**Table:**

Geometry. Bond distances, angles etc. have been calculated using the rounded fractional coordinates. All su's are estimated from the variances of the (full) variance-covariance matrix. The cell esds are taken into account in the estimation of distances, angles and torsion angles
Refinement. Refinement of *F*^2^ against ALL reflections. The weighted *R*-factor wR and goodness of fit *S* are based on *F*^2^, conventional *R*-factors *R* are based on *F*, with *F* set to zero for negative *F*^2^. The threshold expression of *F*^2^ > σ(*F*^2^) is used only for calculating *R*-factors(gt) etc. and is not relevant to the choice of reflections for refinement. *R*-factors based on *F*^2^ are statistically about twice as large as those based on *F*, and *R*- factors based on ALL data will be even larger.

### Fractional atomic coordinates and isotropic or equivalent isotropic displacement parameters (Å^2^)

**Table:**

	*x*	*y*	*z*	*U*_iso_*/*U*_eq_	
O41	0.59084 (14)	0.14581 (11)	0.23776 (10)	0.0489 (4)	
O42	0.42667 (14)	0.05808 (12)	0.17918 (10)	0.0510 (4)	
N1	0.16696 (14)	0.34798 (12)	0.86559 (10)	0.0324 (4)	
N41	0.46108 (16)	0.11510 (12)	0.24364 (11)	0.0383 (4)	
C2	0.23613 (17)	0.20902 (15)	0.88611 (13)	0.0337 (4)	
C3	0.18362 (17)	0.16091 (14)	0.80647 (12)	0.0320 (4)	
C4	0.05789 (16)	0.25761 (14)	0.70081 (12)	0.0289 (4)	
C5	−0.01214 (17)	0.40136 (14)	0.68153 (13)	0.0316 (4)	
C6	0.04413 (18)	0.44346 (15)	0.76571 (13)	0.0346 (4)	
C11	0.12505 (16)	0.18753 (13)	0.51267 (12)	0.0283 (4)	
C21	0.28151 (17)	0.20737 (14)	0.51098 (12)	0.0308 (4)	
C31	0.39116 (17)	0.18636 (14)	0.42196 (12)	0.0319 (4)	
C41	0.34282 (17)	0.14340 (13)	0.33503 (12)	0.0308 (4)	
C42	0.00262 (17)	0.20617 (16)	0.61020 (13)	0.0363 (5)	
C51	0.18791 (18)	0.12374 (14)	0.33259 (13)	0.0360 (4)	
C61	0.07906 (17)	0.14782 (14)	0.42063 (13)	0.0342 (4)	
O2A	0.08063 (12)	0.85096 (11)	0.92983 (10)	0.0424 (3)	
O11A	0.28861 (12)	0.41609 (10)	1.02623 (9)	0.0375 (3)	
O12A	0.11745 (14)	0.63753 (11)	0.88914 (10)	0.0477 (4)	
O51A	0.63063 (13)	0.55882 (12)	1.41523 (10)	0.0484 (4)	
O52A	0.65275 (13)	0.37091 (12)	1.38388 (11)	0.0512 (4)	
N5A	0.58706 (15)	0.50136 (14)	1.35463 (11)	0.0362 (4)	
C1A	0.28307 (16)	0.61579 (14)	1.06572 (12)	0.0282 (4)	
C2A	0.20276 (17)	0.76553 (14)	1.03229 (13)	0.0318 (4)	
C3A	0.24883 (18)	0.82552 (15)	1.10787 (13)	0.0363 (4)	
C4A	0.37383 (17)	0.73914 (15)	1.21296 (13)	0.0346 (5)	
C5A	0.45327 (16)	0.59222 (14)	1.24384 (12)	0.0303 (4)	
C6A	0.40963 (16)	0.53007 (14)	1.17166 (12)	0.0279 (4)	
C11A	0.22703 (17)	0.55144 (15)	0.98847 (13)	0.0316 (4)	
H1	0.213 (2)	0.3733 (19)	0.9259 (18)	0.074 (6)*	
H2	0.32160	0.14400	0.95590	0.0400*	
H3	0.23190	0.06350	0.82290	0.0380*	
H5	−0.09690	0.46890	0.61180	0.0380*	
H6	−0.00380	0.53970	0.75310	0.0420*	
H21	0.31330	0.23530	0.57090	0.0370*	
H31	0.49520	0.20090	0.42090	0.0380*	
H51	0.15750	0.09490	0.27290	0.0430*	
H61	−0.02710	0.13740	0.41850	0.0410*	
H421	−0.10870	0.27540	0.56260	0.0440*	
H422	−0.01130	0.11370	0.66220	0.0440*	
H2A	0.078 (3)	0.773 (2)	0.9050 (19)	0.091 (7)*	
H3A	0.19460	0.92380	1.08670	0.0440*	
H4A	0.40530	0.77840	1.26320	0.0420*	
H6A	0.46480	0.43160	1.19400	0.0340*	

### Atomic displacement parameters (Å^2^)

**Table:**

	*U*^11^	*U*^22^	*U*^33^	*U*^12^	*U*^13^	*U*^23^
O41	0.0475 (7)	0.0522 (7)	0.0479 (7)	−0.0139 (5)	0.0127 (5)	−0.0259 (5)
O42	0.0613 (7)	0.0543 (7)	0.0382 (6)	−0.0051 (5)	−0.0024 (5)	−0.0320 (5)
N1	0.0377 (6)	0.0375 (7)	0.0289 (6)	−0.0167 (5)	0.0056 (5)	−0.0181 (5)
N41	0.0443 (7)	0.0313 (6)	0.0287 (6)	−0.0011 (6)	−0.0020 (5)	−0.0124 (5)
C2	0.0347 (7)	0.0353 (8)	0.0267 (7)	−0.0090 (6)	−0.0010 (6)	−0.0118 (6)
C3	0.0368 (8)	0.0297 (7)	0.0299 (7)	−0.0101 (6)	0.0025 (6)	−0.0145 (6)
C4	0.0295 (7)	0.0364 (7)	0.0256 (7)	−0.0153 (6)	0.0062 (5)	−0.0153 (6)
C5	0.0303 (7)	0.0326 (7)	0.0279 (7)	−0.0082 (6)	0.0009 (5)	−0.0115 (6)
C6	0.0394 (8)	0.0304 (7)	0.0346 (8)	−0.0123 (6)	0.0076 (6)	−0.0149 (6)
C11	0.0344 (7)	0.0232 (7)	0.0253 (7)	−0.0088 (6)	−0.0029 (5)	−0.0093 (5)
C21	0.0395 (8)	0.0313 (7)	0.0274 (7)	−0.0154 (6)	0.0006 (6)	−0.0155 (6)
C31	0.0362 (7)	0.0320 (7)	0.0292 (7)	−0.0137 (6)	0.0011 (6)	−0.0134 (6)
C41	0.0375 (7)	0.0241 (7)	0.0245 (7)	−0.0033 (6)	−0.0016 (6)	−0.0103 (5)
C42	0.0364 (8)	0.0465 (9)	0.0350 (8)	−0.0190 (7)	0.0028 (6)	−0.0226 (7)
C51	0.0428 (8)	0.0354 (8)	0.0318 (7)	−0.0081 (6)	−0.0080 (6)	−0.0199 (6)
C61	0.0343 (8)	0.0350 (7)	0.0352 (7)	−0.0115 (6)	−0.0047 (6)	−0.0171 (6)
O2A	0.0421 (6)	0.0345 (6)	0.0410 (6)	−0.0050 (5)	−0.0063 (5)	−0.0134 (5)
O11A	0.0435 (6)	0.0341 (6)	0.0381 (6)	−0.0102 (4)	−0.0051 (4)	−0.0205 (5)
O12A	0.0556 (7)	0.0421 (6)	0.0397 (6)	−0.0083 (5)	−0.0168 (5)	−0.0181 (5)
O51A	0.0545 (7)	0.0607 (7)	0.0454 (6)	−0.0312 (6)	−0.0048 (5)	−0.0280 (5)
O52A	0.0475 (6)	0.0438 (6)	0.0574 (7)	−0.0075 (5)	−0.0177 (5)	−0.0226 (6)
N5A	0.0347 (6)	0.0464 (8)	0.0363 (7)	−0.0208 (6)	0.0015 (5)	−0.0210 (6)
C1A	0.0273 (7)	0.0331 (7)	0.0287 (7)	−0.0131 (6)	0.0066 (5)	−0.0160 (6)
C2A	0.0299 (7)	0.0330 (7)	0.0321 (7)	−0.0115 (6)	0.0056 (6)	−0.0136 (6)
C3A	0.0403 (8)	0.0310 (7)	0.0426 (8)	−0.0146 (6)	0.0090 (6)	−0.0193 (7)
C4A	0.0385 (8)	0.0409 (8)	0.0385 (8)	−0.0217 (7)	0.0100 (6)	−0.0245 (7)
C5A	0.0272 (7)	0.0379 (8)	0.0314 (7)	−0.0153 (6)	0.0041 (5)	−0.0174 (6)
C6A	0.0271 (7)	0.0307 (7)	0.0296 (7)	−0.0122 (6)	0.0042 (5)	−0.0149 (6)
C11A	0.0317 (7)	0.0382 (8)	0.0286 (7)	−0.0131 (6)	0.0040 (6)	−0.0173 (6)

### Geometric parameters (Å, °)

**Table:**

O41—N41	1.2244 (19)	C41—C51	1.379 (2)
O42—N41	1.2300 (18)	C51—C61	1.380 (2)
O2A—C2A	1.3378 (18)	C2—H2	0.9300
O11A—C11A	1.256 (2)	C3—H3	0.9300
O12A—C11A	1.2613 (18)	C5—H5	0.9300
O51A—N5A	1.2331 (19)	C6—H6	0.9300
O52A—N5A	1.227 (2)	C21—H21	0.9300
O2A—H2A	1.00 (2)	C31—H31	0.9300
N1—C6	1.3366 (18)	C42—H421	0.9700
N1—C2	1.336 (2)	C42—H422	0.9700
N41—C41	1.4673 (19)	C51—H51	0.9300
N1—H1	0.97 (2)	C61—H61	0.9300
N5A—C5A	1.4536 (18)	C1A—C6A	1.3828 (19)
C2—C3	1.363 (2)	C1A—C11A	1.494 (2)
C3—C4	1.3884 (19)	C1A—C2A	1.412 (2)
C4—C42	1.506 (2)	C2A—C3A	1.402 (2)
C4—C5	1.387 (2)	C3A—C4A	1.370 (2)
C5—C6	1.372 (2)	C4A—C5A	1.390 (2)
C11—C61	1.394 (2)	C5A—C6A	1.380 (2)
C11—C21	1.388 (2)	C3A—H3A	0.9300
C11—C42	1.516 (2)	C4A—H4A	0.9300
C21—C31	1.383 (2)	C6A—H6A	0.9300
C31—C41	1.376 (2)		
			
C2A—O2A—H2A	97.0 (12)	C5—C6—H6	120.00
C2—N1—C6	120.47 (13)	C11—C21—H21	119.00
O41—N41—C41	118.56 (12)	C31—C21—H21	119.00
O42—N41—C41	118.07 (13)	C41—C31—H31	121.00
O41—N41—O42	123.34 (13)	C21—C31—H31	121.00
C6—N1—H1	123.7 (12)	C4—C42—H421	109.00
C2—N1—H1	115.8 (12)	C4—C42—H422	109.00
O52A—N5A—C5A	118.88 (13)	C11—C42—H421	109.00
O51A—N5A—O52A	122.56 (13)	C11—C42—H422	109.00
O51A—N5A—C5A	118.56 (14)	H421—C42—H422	108.00
N1—C2—C3	121.14 (13)	C61—C51—H51	121.00
C2—C3—C4	119.88 (14)	C41—C51—H51	121.00
C3—C4—C5	117.93 (13)	C11—C61—H61	119.00
C5—C4—C42	121.87 (12)	C51—C61—H61	119.00
C3—C4—C42	120.20 (14)	C2A—C1A—C11A	119.62 (12)
C4—C5—C6	119.72 (13)	C6A—C1A—C11A	120.95 (14)
N1—C6—C5	120.84 (15)	C2A—C1A—C6A	119.41 (13)
C21—C11—C61	118.29 (13)	O2A—C2A—C1A	120.58 (13)
C42—C11—C61	118.58 (13)	O2A—C2A—C3A	119.38 (14)
C21—C11—C42	123.13 (12)	C1A—C2A—C3A	120.04 (13)
C11—C21—C31	121.31 (13)	C2A—C3A—C4A	119.91 (15)
C21—C31—C41	118.51 (14)	C3A—C4A—C5A	119.44 (14)
N41—C41—C31	119.00 (13)	N5A—C5A—C6A	118.90 (14)
C31—C41—C51	122.04 (13)	C4A—C5A—C6A	121.88 (13)
N41—C41—C51	118.94 (12)	N5A—C5A—C4A	119.22 (13)
C4—C42—C11	114.93 (12)	C1A—C6A—C5A	119.31 (14)
C41—C51—C61	118.52 (13)	O11A—C11A—C1A	118.90 (12)
C11—C61—C51	121.28 (14)	O12A—C11A—C1A	117.35 (14)
N1—C2—H2	119.00	O11A—C11A—O12A	123.74 (14)
C3—C2—H2	119.00	C2A—C3A—H3A	120.00
C4—C3—H3	120.00	C4A—C3A—H3A	120.00
C2—C3—H3	120.00	C3A—C4A—H4A	120.00
C4—C5—H5	120.00	C5A—C4A—H4A	120.00
C6—C5—H5	120.00	C1A—C6A—H6A	120.00
N1—C6—H6	120.00	C5A—C6A—H6A	120.00
			
C6—N1—C2—C3	−0.1 (2)	C11—C21—C31—C41	−0.7 (2)
C2—N1—C6—C5	−0.7 (2)	C21—C31—C41—N41	−177.57 (13)
O41—N41—C41—C31	−7.89 (19)	C21—C31—C41—C51	1.3 (2)
O41—N41—C41—C51	173.20 (13)	C31—C41—C51—C61	−0.2 (2)
O42—N41—C41—C31	170.27 (13)	N41—C41—C51—C61	178.73 (13)
O42—N41—C41—C51	−8.65 (19)	C41—C51—C61—C11	−1.6 (2)
O52A—N5A—C5A—C4A	177.68 (13)	C6A—C1A—C2A—O2A	179.64 (13)
O52A—N5A—C5A—C6A	−2.8 (2)	C6A—C1A—C2A—C3A	−1.4 (2)
O51A—N5A—C5A—C4A	−2.7 (2)	C11A—C1A—C2A—O2A	−2.0 (2)
O51A—N5A—C5A—C6A	176.89 (13)	C11A—C1A—C2A—C3A	177.02 (13)
N1—C2—C3—C4	1.0 (2)	C2A—C1A—C6A—C5A	0.9 (2)
C2—C3—C4—C42	178.32 (13)	C11A—C1A—C6A—C5A	−177.44 (13)
C2—C3—C4—C5	−1.0 (2)	C2A—C1A—C11A—O11A	−173.72 (13)
C3—C4—C5—C6	0.2 (2)	C2A—C1A—C11A—O12A	5.3 (2)
C42—C4—C5—C6	−179.08 (13)	C6A—C1A—C11A—O11A	4.6 (2)
C3—C4—C42—C11	−77.72 (17)	C6A—C1A—C11A—O12A	−176.38 (13)
C5—C4—C42—C11	101.54 (17)	O2A—C2A—C3A—C4A	−179.94 (14)
C4—C5—C6—N1	0.6 (2)	C1A—C2A—C3A—C4A	1.1 (2)
C61—C11—C21—C31	−1.0 (2)	C2A—C3A—C4A—C5A	−0.3 (2)
C21—C11—C42—C4	3.8 (2)	C3A—C4A—C5A—N5A	179.37 (13)
C61—C11—C42—C4	−176.59 (13)	C3A—C4A—C5A—C6A	−0.2 (2)
C21—C11—C61—C51	2.2 (2)	N5A—C5A—C6A—C1A	−179.70 (12)
C42—C11—C21—C31	178.66 (14)	C4A—C5A—C6A—C1A	−0.2 (2)
C42—C11—C61—C51	−177.47 (14)		

### Hydrogen-bond geometry (Å, °)

**Table:**

*D*—H···*A*	*D*—H	H···*A*	*D*···*A*	*D*—H···*A*
N1—H1···O11A	0.97 (2)	1.612 (19)	2.5804 (16)	180 (2)
N1—H1···O12A	0.97 (2)	2.55 (2)	3.1464 (18)	120.2 (14)
O2A—H2A···O12A	1.00 (2)	1.48 (2)	2.4623 (17)	165 (2)
C2—H2···O42^i^	0.93	2.39	3.2153 (17)	148
C6—H6···O12A	0.93	2.59	3.185 (2)	122
C21—H21···O51A^ii^	0.93	2.49	3.271 (2)	142
C31—H31···O52A^iii^	0.93	2.49	3.322 (2)	149
C42—H421···O52A^iv^	0.97	2.50	3.3828 (19)	151

Symmetry codes: (i) *x*, *y*, *z*+1; (ii) −*x*+1, −*y*+1, −*z*+2; (iii) *x*, *y*, *z*−1; (iv) *x*−1, *y*, *z*−1.
